# Clinical versus fixed warfarin dosing and the impact on quality of anticoagulation (The ClinFix trial)

**DOI:** 10.1111/cts.13797

**Published:** 2024-06-10

**Authors:** Amr M. Fahmi, Ahmed El Bardissy, Mohamed Omar Saad, Mohamed Nabil Elshafei, Loulia Bader, Ahmed Mahfouz, Mohamed Kasem, Osama Abdelsamad, Abdelnasser Elzouki, Christina L. Aquilante, Fatima Mraiche, Ezeldin Soaly, Ihab El Madhoun, Nidal Asaad, Abdulrahman Arabi, Eman Alhmoud, Hazem Elewa

**Affiliations:** ^1^ Pharmacy Department Hamad Medical Corporation Doha Qatar; ^2^ College of Pharmacy Qatar University Doha Qatar; ^3^ Department of Medicine, Hamad General Hospital Hamad Medical Corporation Doha Qatar; ^4^ Department of Pharmaceutical Sciences Skaggs School of Pharmacy and Pharmaceutical Sciences, University of Colorado Aurora USA; ^5^ Department of Pharmacology, Faculty of Medicine and Dentistry University of Alberta Edmonton Alberta Canada; ^6^ Department of Cardiology AlWakra Hospital, Hamad Medical Corporation AlWakra Qatar; ^7^ Department of Medicine AlWakra Hospital, Hamad Medical Corporation AlWakra Qatar; ^8^ Department of Cardiology Heart Hospital, Hamad Medical Corporation Doha Qatar

## Abstract

Different dosing strategies exist to initiate warfarin, most commonly fixed warfarin dosing (FWD), clinical warfarin dosing (CWD), and genetic‐guided warfarin dosing (GWD). Landmark trials have shown GWD to be superior when compared to FWD in the EU‐PACT trial or CWD in the GIFT trial. COAG trial did not show differences between GWD and CWD. We aim to compare the anticoagulation quality outcomes of CWD and FWD. This is a prospective cohort study with a retrospective comparator. Recruited subjects in the CWD (prospective) arm were initiated on warfarin according to the clinical dosing component of the algorithm published in www.warfarindosing.org. The primary efficacy outcome was the percentage time in the therapeutic range (PTTR) from day 3 to 6 till day 28 to 35. The study enrolled 122 and 123 patients in the CWD and FWD, respectively. The PTTR did not differ statistically between CWD and FWD (62.2 ± 26.2% vs. 58 ± 25.4%, *p* = 0.2). There was also no difference between both arms in the percentage of visits with extreme subtherapeutic international normalized ratio (INR) (<1.5; 15 ± 18.3% vs. 16.8 ± 19.1%, *p* = 0.44) or extreme supratherapeutic INR (>4; 7.7 ± 14.7% vs. 7.5 ± 12.4%, *p* = 0.92). We conclude that CWD did not improve the anticoagulation quality parameters compared to the FWD method.

**Abbreviations**
COAG trial: The Clarification of Optimal Anticoagulation through Genetics
CWD: clinical warfarin dosing
CYP: cytochrome P 450
DOACs: direct oral anticoagulants
EU‐PACT trial: The European Pharmacogenetics of Anticoagulant Therapy trial
FWD: fixed warfarin dosing
GIFT trial: The Genetic Informatics trial
GWD: genetic‐guided warfarin dosing
INR: international normalized ratio
PTTR: percentage of time in therapeutic range
SD: standard deviation
VKORC1: Vitamin k epoxide reductase complex 1

**Study Highlights**

**WHAT IS THE CURRENT KNOWLEDGE ON THE TOPIC?**

GWD had better outcomes when compared to FWD, whereas GWD produced mixed outcomes when compared to CWD in two studies, in one of which there were no differences in the outcomes (COAG trial), whereas in the second study, the GWD had better outcomes when compared with CWD (GIFT trial). No clinical studies to date have compared FWD and CWD.

**WHAT QUESTION DID THIS STUDY ADDRESS?**

Is CWD better than FWD in terms of anticoagulation quality during warfarin initiation?

**WHAT DOES THIS STUDY ADD TO OUR KNOWLEDGE?**

No differences exist between CWD and FWD in terms of the anticoagulation quality outcomes such as PTTR.

**HOW MIGHT THIS CHANGE CLINICAL PHARMACOLOGY OR TRANSLATIONAL SCIENCE?**

Since no differences exist between the two arms, this could add some evidence to explain why there was controversy in the studies that compared GWD and CWD and focus more on other reasons like the mixed population in the COAG trial.

## INTRODUCTION

Warfarin prescribing has always been a source of challenge to different healthcare professionals.^1^ While direct oral anticoagulants (DOACs) are the anticoagulant of choice for the majority of patients,^2^, ^3^ warfarin remains prescribed in a number of indications where DOACs failed to show non‐inferiority to warfarin.^4^ A number of methods exist for warfarin initiation, the most common are fixed warfarin dosing (FWD), clinical warfarin dosing (CWD), and genetic‐guided warfarin dosing (GWD).^5^ FWD involves administering an empirical warfarin dose for 1–2 days while checking the international normalized ratio (INR) on the 3rd day and adjusting warfarin dose accordingly. CWD involves the use of patients' clinical factors and incorporating these factors into a clinical algorithm which recommends a warfarin initiation dose. Examples of these clinical factors include age, sex, race, interacting medications, liver impairment, baseline INR, and indication for anticoagulation. GWD uses patients' genetic information pertaining to warfarin pharmacology, in addition to clinical factors, to recommend a warfarin initiation dose. Examples of relevant warfarin genetic testing results include *VKORC1 c.‐1639G> A* (rs9923231), CYP2C9*2 (rs1799853), CYP2C9*3 (rs1057910), and CYP4F2*3 (rs2108622) variants.^6^

In the last decade, three large clinical trials compared GWD to either FWD or CWD. In the EU‐PACT trial, GWD was compared to FWD in a predominantly European population and was found to be superior in terms of the percentage of time in therapeutic range (PTTR; 67.4% in the GWD vs. 60.3% in the FWD, adjusted difference, 7% [95% CI, 3.3 to 10.6], *p* < 0.001).^7^ The COAG trial, on the other hand, compared GWD to CWD in a racially mixed population (27% were of non‐European descent) and found no difference in terms of PTTR between the two arms (45.2% in the GWD and 45.4% in the CWD, adjusted mean difference, −0.2%, [95% CI, −3.4 to 3.1], *p* = 0.91).^8^ The GIFT trial, which was the most recent among the three studies, compared GWD to CWD in a primarily European population with a primary composite outcome of major bleeding, INR of 4 or greater, venous thromboembolism, or death.^9^ The primary composite outcome was found to be improved in the GWD compared to the CWD (10.8% vs. 14.7%, absolute difference, 3.9% [95% CI, 0.7–7.2], *p* = 0.02).

Some experts attributed the contrast in the outcomes between these three trials to the mixed population used in the COAG trial compared to the EU‐PACT or the GIFT trials.^10^ The genetic variants that affect warfarin metabolism differ between the European population and the African American population (mainly coming from West Africa).^6^ African populations have other variants that were not tested in the COAG trial (example*: CYP2C* cluster [rs12777823], CYP2C9*5 [rs28371686], *6 [rs9332131], *8 [rs7900194], and *11 [rs28371685]). This may have led to the negative results seen with the GWD in the COAG trial whereas this was not the case in the EU‐PACT and GIFT trials since they included a more homogenous population, primarily of European descent. Another proposed reason for the differences in the results between the three trials is the use of different comparator arms (FWD vs. CWD). In fact, previous non‐clinical studies have suggested that CWD has better outcomes when compared to FWD in terms of the ability to predict the actual warfarin maintenance dose or the mean absolute percentage error.^11^ However, to date, no clinical studies have compared CWD to FWD. In the ClinFix trial, the aim was to compare CWD to FWD to determine the best warfarin dosing initiation strategy when GWD is not available. Additionally, the study sought to provide input on possible reasons for the differences in the outcomes between the abovementioned trials.

## METHODS

### Study design and settings

The ClinFix trial was a pragmatic multicenter study designed to test the superiority of CWD compared to FWD in terms of anticoagulation quality and clinical outcomes. The protocol was approved by the Medical Research Centre in Hamad Medical Corporation (HMC), Doha, Qatar and the institutional review board, Qatar University, Doha, Qatar. Patients in the CWD were recruited prospectively and were compared to a retrospective (historical) FWD cohort. CWD patients were recruited from three different hospitals in Qatar; Al Wakra Hospital, Hamad General Hospital, and Heart Hospital. These hospitals are part of HMC, which is the leading health provider in Qatar. Patients recruited in the CWD arm and collected data for the FWD cohort had to have at least two INR readings in the first week and then weekly thereafter for a total of 4 weeks. Patients were followed up for at least 3 months after warfarin initiation. Follow‐up was performed at the respective hospital anticoagulation clinic.

### Participants

#### CWD arm

Eligible patients met the following criteria: 18 years or older, were able to follow‐up with outpatient anticoagulation clinic, had a planned warfarin duration of at least 3 months, were of Arab descent, received no more than one dose of warfarin at the time of the consent, and prescribers agreed to adhere to the provided clinical dosing algorithm for at least three (preferably five) days.

Patients were excluded if: had prior therapy with known warfarin maintenance dose, prescriber chose not to use CWD for reasons not accounted for in the algorithm, abnormal baseline INR (>1.45), life expectancy of <1 year, patient factors that may lead to non‐adherence to warfarin or anticoagulation clinic visits (e.g., dementia).

All eligible patients were recruited and initiated on warfarin using the CWD strategy between October 2020 and June 2023. The CWD was calculated using the Gage et al.^12^ algorithm available online at “www.warfarindosing.org.” The algorithm is primarily used for GWD, but it allows for CWD calculations if genetic results are not available. Patients in the CWD arm were also asked to participate in a genetic sub‐study and provide a saliva sample for future genetic analysis.

Once the patient consented to the CWD study, the initial dose of warfarin was calculated using the Gage et al. algorithm. Patients were started on the algorithm‐calculated recommended dose. An adjustment of 0.5 mg in the recommended maintenance warfarin dose was allowed for doses that were more than 3 mg daily. Also, loading doses (for the first 1–2 days) and dose revisions (after 3–5 days from initiation) were allowed and dose titration thereafter was performed at the physician's discretion. Patients' INR levels were followed up during hospitalization and in outpatient anticoagulation clinics for at least 4 weeks and clinical outcomes were monitored for 3 months.

#### FWD arm (retrospective, historical cohort)

Patients initiated on warfarin between January 2016 and January 2020 were screened for eligibility. Retrospective data for patients meeting inclusion/exclusion criteria were extracted from the medical record. Since CWD was used as routine practice in Al Wakra Hospital since Jan 2019, we included patients' data from January 2016 to December 2017 from Al Wakra Hospital and from January 2018 to December 2019 from Hamad General Hospital and Heart Hospital. Patients included in the FWD retrospective cohort were confirmed to have at least two INR results during the first week of initiation and weekly thereafter for a total of 4 weeks. Additionally, patients were confirmed to have regular follow‐up at one of the HMC anticoagulation clinics, with full reported information in the electronic medical record used at HMC (Cerner) for a minimum of 3 months after initiation. All patients' data meeting the inclusion/exclusion criteria were utilized.

### Study outcomes

The primary outcome was the difference in the percentage of time patients spent in therapeutic range (PTTR) between the CWD and FWD arms which was calculated starting from day^3^, ^4^, ^5^, ^6^ of warfarin and for a total of 4 weeks. If INR was not available on the 28th day, INR could be obtained up to day 35. Patients who stopped warfarin for more than five consecutive days were excluded from the analysis. PTTR was calculated using the Rosendaal formula, which assumes linear INR changes between visits.^13^ Other outcomes obtained during the follow‐up period included the percentage of visits within the therapeutic range, the percentage of extreme subtherapeutic INRs (INR < 1.5), and the percentage of extreme supratherapeutic INR (INR > 4).

Clinical outcomes were followed up for 3 months after initiation. These outcomes included (1) thromboembolic events, which were defined as the occurrence of any of the following: deep vein thrombosis (DVT) confirmed by ultrasound or computed tomography (CT) angiogram; pulmonary embolism (PE) confirmed by CT pulmonary angiography; embolic stroke confirmed by clinical examination and CT or magnetic resonance imaging of the brain. (2) Bleeding events which were classified according to the International Society of Thrombosis and Hemostasis guidelines as either major or non‐major bleeding.^14^ Major bleeding was defined as having any of the following: bleeding and hemodynamic instability; bleeding in a critical site; hemoglobin drop of more than 2 g/dL, or if the patient received two units of packed red blood cells. Non‐major bleeding was defined as any bleeding that did not fit the criteria for major bleeding. Anticoagulation‐related emergency room (ER) visits and anticoagulation‐related hospitalization were defined as any ER visit or hospitalization which was directly related to oral anticoagulant therapy, respectively.

### Statistical analysis and sample size calculation

Baseline characteristics and study outcomes of the two arms were compared using Student *t*‐test for continuous variables whereas Chi‐square and Fisher exact tests were used for categorical variables. Data were presented as mean ± standard deviation (SD) or frequencies and percentages. To exclude the presence of confounding variables associated with PTTR, univariate regression analysis was performed. Variables with a *p*‐value <0.2 were included thereafter in a multivariable regression model. Multivariable regression analyses of these variables were executed to explore any significant effects on the dependent variable (PTTR). Variables with a *p*‐value >0.2 were removed. IBM SPSS statistics 25 program was used for statistical analysis.

The sample size was calculated using the University of Carolina San Francisco T statistic sample size calculator (from www.data.ucsf.edu/research/sample‐size). A sample size of 115 subjects per arm was derived based on a standard deviation of 27%, used in previous research,^7^ to detect a 10% difference in the PTTR between the two arms with a power of 80% and an alpha of 0.05. We aimed to include 140 subjects per arm to account for a possible 20% dropout rate in the CWD arm.

## RESULTS

### Participants

**FIGURE 1:**
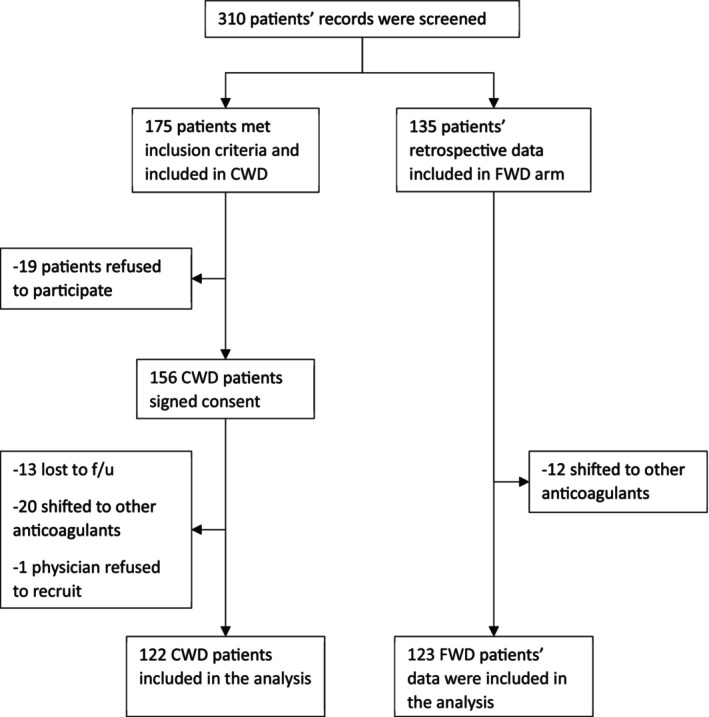
Patients' recruitment flow chart. CWD, clinical warfarin dosing; FWD, fixed warfarin dosing.

In both arms, out of the 310 patients screened, nearly 20% were excluded due to various reasons (Figure 1). A total of 245 subjects and subjects' data were included in the analysis, 122 from the CWD arm and 123 from the FWD arm. The baseline characteristics are shown in Table 1. The study had an almost equal distribution of men (55%) and women (45%) and the mean age was 52.9 ± 16.6 years. Most of the patients (65.5%) received warfarin for either DVT, PE, or atrial fibrillation (AF). Most of the patients (99.4%) were from an Arab country with the majority being from Qatar and Egypt (50.6%). Patients in the CWD arm had a significantly higher body weight than patients in the FWD arm (92.8 ± 24.9 kg vs. 86.2 ± 25.9 kg, *p* = 0.04), but body mass index did not differ statistically between the groups (32.6 ± 8.2 kg/m^2^ vs. 31.9 ± 8.62 kg/m^2,^, respectively, *p* = 0.52). Additionally, there were significantly more males in the CWD versus FWD arm (79 [65.6%] vs. 55 [44.7%], *p* = 0.001) and more patients received a loading dose in the CWD than in the FWD arm (45 [37.5%] vs. 21 [17.1%], *p* < 0.001). On the other hand, the FWD arm had a significantly greater proportion of patients with coronary artery diseases than the CWD cohort (15 [12.5%] vs. 33 [26.8%], *p* = 0.005). The mean warfarin dose was similar in both arms (4.8 ± 2.4 mg per day in the CWD vs. 4.9 ± 2.4 mg per day, 95% CI: −0.698 to 0.594).

**TABLE 1:** Baseline characteristics.

Baseline characteristics	CWD (*N* = 122)	FWD (*N* = 123)	*p*‐value
Age (years) – mean ± SD	52.5 ± 14.7	53.7 ± 18.5	0.56
Weight (kg) – mean ± SD	92.5 ± 24.9	86.2 ± 25.9	0.05
Height (cm) –mean ± SD	168.2 ± 8.9	163.9 ± 9.8	<0.001
Body mass index (kg/m^2^)‐mean ± SD	32.6 ± 8.2	31.9 ± 8.6	0.53
Male (%)	79 (65.6)	55 (44.7)	0.001
Blacks (%)	12 (10)	16 (13)	0.46
Origin (%)			
GCC & Yemen	41 (33.6)	39 (31.7)	0.35
Levant	39 (32)	32 (26)	
Egypt	26 (21.3)	26 (21.1)	
Sudan	11 (9)	16 (13)	
North Africa	2 (1.6)	7 (5.7)	
Other^a^	3 (2.4)	1 (0.8)	
Indication for anticoagulation (%)			
AF	22 (18)	37 (30.1)	0.001
DVT	25 (20.5)	34 (27.6)	
PE	19 (15.6)	23 (18.7)	
Prosthetic valves	5 (4.1)	3 (2.4)	
LVT	19 (15.6)	2 (1.6)	
Other^b^	32 (26.2)	24 (19.5)	
Diabetes (%)	52 (42.6)	42 (34.1)	0.17
Hypertension (%)	65 (53.3)	61 (49.6)	0.56
Heart failure (%)	16 (13.1)	24 (19.5)	0.18
Coronary artery diseases (%)	15 (12.3)	33 (26.8)	0.004
Liver diseases (%)	2 (1.6)	0 (0)	0.15
Patients who smoke (%)	30 (24.6)	12 (9.8)	0.002
Patients who drink alcohol (%)	2 (1.7)	0 (0)	0.15
Interacting medications (%)^c^	13 (10.8)	15 (12.2)	0.7
Target INR other than 2–3 (%)	0 (0)	2 (1.6)	0.16
Anticoagulation duration (%)			
3 months	23 (28.9)	13 (10.6)	0.2
6 months	26 (21.3)	26 (21.1)	
Undefined	17 (13.9)	14 (11.4)	
Life long	56 (45.9)	70 (56.9)	
Baseline INR‐mean ± SD	1.1 ± 0.1	1.1 ± 0.1	0.94
Received loading dose (%)	45 (36.9)	21 (17.1)	<0.001
Daily warfarin dose (mg) – mean ± SD	4.8 ± 2.4	4.9 ± 2.4	0.87
Total warfarin days For TTR calculation‐mean ± SD	31.8 ± 7.1	30.6 ± 5.1	0.11
Initial day for TTR calculation – mean ± SD	4.6 ± 0 0.96	4.8 ± 0.85	0.07
Number of visits – mean ± SD	9.1 ± 4.9	9.3 ± 4.5	0.93

Abbreviatons: AF, atrial fibrillation; DVT, deep vein thrombosis; GCC, Gulf council countries; INR, international normalized ratio; PE, pulmonary embolism; LV, left ventricular thrombus; SD, standard deviation; TTR, time in therapeutic range.

^a^ Other: Iraq, India.

^b^ Other: Cerebral vein thrombosis, portal vein thrombosis, mesenteric vein thrombosis, hepatic vein thrombosis, jugular vein thrombosis, renal vein thrombosis, peripheral vascular disease, LV non‐compaction, arterio‐venous fistula thrombosis, and atrial thrombosis).

^c^ Interacting medications: Any medication that interacts with warfarin and has a category higher than category C according to Lexicomp® interaction checker. (See Table S1 for details of interacting medication and their interaction category).

### Outcomes

**FIGURE 2:**
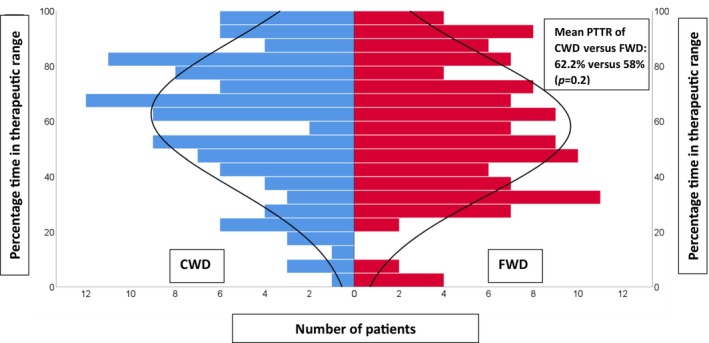
Distribution of PTTR of the CWD (in blue) arranged side by side with FWD (in red) from days 3–6 to days 28–35. CWD, clinical warfarin dosing; FWD, fixed warfarin dosing.

The overall mean PTTR for the first month after the initiation phase (from days 3–6 to days 28–35) was (60.1 ± 25.8%). The average number of days used for PTTR calculation was (30.5 ± 6), and the follow‐up duration for clinical outcomes was 3 months for all patients included in the study. There were no differences in the primary outcome of PTTR between the CWD (62.2 ± 26.2%) and FWD (58 ± 25.4%) arms with an absolute mean difference of 4.2% (95% CI: −2.2 to 10.7%, *p* = 0.2; Figure 2). All variables were tested using univariate regression analysis to examine their effect on PTTR and each variable with a *p*‐value <0.2 was included thereafter in a multivariable regression analysis. Only three variables were identified as possible confounders; having coronary artery diseases, having heart failure, and co‐prescribing of interacting medications (Table S2). The results of the multivariable model showed no significant effect from these variables on the primary outcome. Secondary outcomes, including clinical outcomes, showed no statistical difference between the CWD and FWD arms (Table 2).

**TABLE 2:** Outcomes.

Outcomes	CWD (*N* = 120)	FWD (*N* = 123)	*p*‐value
Anticoagulation quality outcomes (1 month) mean ± SD			
Percentage time in therapeutic range	62.2% ± 26.2	58% ± 25.4	0.2
Percentage of visits within range	57.7% ± 23	55.7% ± 22.7	0.6
Days in range‐ mean ± SD	20.1 ± 10.2	17.9 ± 8.7	0.07
Days to first therapeutic INR	7.8 ± 8.6	8.2 ± 8.3	0.73
Days to stable warfarin dose	25.5 ± 27.5	23.9 ± 23.4	0.64
Percentage of extreme subtherapeutic INR	15% ± 18.3	16.8% ± 19.1	0.44
Percentage of extreme supratherapeutic INR	7.7 (± 14.7)	7.5 (± 12.4)	0.92
Patients with no stable dose within 3 months	14 (11.5)	15 (12.2)	0.86
Clinical outcomes (3 months) number of events (cases per 100 persons month)			
Major bleeding	4 (3.3)	2 (1.6)	0.4
Non‐major bleeding	5 (4.2)	8 (6.5)	0.4
Thromboembolism	3 (2.4)	2 (1.6)	0.6
Anticoagulation‐related ER visits	6 (5)	4 (3.3)	0.47
Anticoagulation‐related hospitalization	3 (2.4)	8 (6.5)	0.24

Abbreviations: CWD, clinical warfarin dosing; FWD, fixed warfarin dosing; INR, international normalized ratio; n, number; PTTR, percentage time in therapeutic range; SD, standard deviation.

## DISCUSSION

This study sought to determine whether initiating warfarin with a CWD strategy would yield better outcomes compared to a FWD strategy. Our results showed that PTTR, other anticoagulation quality measures, and clinical outcomes did not differ between the CWD and FWD strategies. This is the first study to directly compare these two dosing strategies, which makes its results highly clinically relevant despite the lack of significance.

Based on two studies from the International Warfarin Pharmacogenetic Consortium (IWPC) and Kimmel's group looking at clinical and genetics factors affecting warfarin dosing in patients with stable warfarin dosing, CWD had better outcomes in terms of coefficient of determination (*R* ^*2*^) and mean absolute percentage error (MAPE) when compared to FWD.^11^, ^15^ These studies, however, were not designed to explore the impact of each dosing strategy on clinical outcomes and key anticoagulation quality measures like PTTR.

We used the Gage et al. algorithm (www.warfarindosing.org) to calculate CWD in this study. The Gage algorithm includes important clinical factors such as age, weight, race, and interacting medications, among others. While other algorithms included slightly different factors, Gage et al. algorithm remains as one of the most validated and widely adopted algorithms. Age is an important factor which contributes significantly to warfarin dose, as described in early studies.^16^, ^17^ Findings from Gage et al. showed that patients 70 years and older were more likely to require a lower warfarin dose than those who were younger. Weight gain and therefore a higher body mass index was also associated with higher warfarin dose requirements, specifically those with morbid obesity. Apart from genetic determinants, race also plays an important role.^18^ Those of African descent have been shown to require higher warfarin doses when compared to their counterparts of European descent.^19^ Also, initiating a medication that could interact with warfarin is associated with variation in warfarin dose.^5^ According to that algorithm, it is obvious that clinical factors play a small role (explain 10% of the warfarin dose diversity) when compared with genetic factors which account for 40% or more of warfarin dose variability.^5^, ^20^, ^21^ This may explain our results which showed that CWD is not different than FWD, and that most of the benefit from this algorithm as well as others is likely attributed to the addition of the genetic factors.

The PTTR values attained in our results are somewhat higher but not much different than data from other landmark studies. In the EUPACT trial, the mean PTTR was 60.3% in the comparator FWD arm. Also, CWD arm of the GIFT trial had a mean PTTR of 51.4% during the first 4 weeks. In the COAG trial, those identified as non‐black in the CWD of the COAG trial had a mean PTTR of 46.1% which was the lowest among all these trials. Despite these differences, our overall result which showed the lack of difference between CWD and FWD is very valid especially that both cohorts were managed within the same settings and included not just the PTTR but many other quality and clinical outcomes and were all not different between both arms.

The importance of our study is emphasized in light of the previous results from the large RCTs that compared GWD to non‐GWD (CWD or FWD). Both the EUPACT and the GIFT trials showed better outcomes in the GWD when compared to FWD and CWD, respectively. The only trial that showed non‐superiority of GWD was the COAG trial. It was debated that the differences in the results of the COAG trial and the other trials were due, in part, to the non‐GWD strategies used in the different trials. The use of CWD in the COAG trial was suggested as a better control arm compared to FWD, resulting in no difference between GWD and CWD. Based on our results, it may imply that this was not the case and that both CWD and FWD produce similar PTTR. Another factor that is thought to influence differences in prospective warfarin PGx trials was related to the dosing algorithm used in the COAG trial, which was tailored primarily to those of European descent. However, the COAG trial included a relatively diverse population (27% African Americans) and did not test for genetic variants known to influence warfarin dose requirements in those of African descent (CYP2C9*5, *6, *8, *11, and the *CYP2C* rs12777823).^6^ In fact, in the same study, stratification by race showed that African Americans who were in GWD arm had a significantly lower PTTR compared to those in the CWD arm while this was not the case with their European counterparts. Given that the results of our study showed no difference between CWD and FWD, it may support the notion that the diverse population in the COAG trial, and not the comparator dosing method, was the main contributor to the non‐significant findings in the COAG trial.

Our study has a number of limitations. First, data for the FWD arm were collected retrospectively, which could have introduced bias to our study, especially with unforeseen confounders. We tried to overcome this by adjusting for possible confounders. However, the primary outcome was PTTR, which relies on the collection of objective data (INR) which can be easily tracked in electronic medical records and are not impacted by the temporal effect. Second, although a dose adjustment, using the www.warfarindosing.org algorithm, was allowed at days 3–5, this was not done in all patients in the CWD arm and was left to the discretion of the treating physician. The evidence for this dose adjustment may not be clinically robust.^22^ However, we believe that this allowed for a more pragmatic approach to warfarin dosing. Another limitation is related to the generalizability of our study, as it primarily included individuals of Arab descent. Thus, validation of these results in more diverse population may be warranted in the future. Fourth is the non‐consistent use of loading doses, as more patients in the CWD arm received loading doses than in the FWD cohort. However, PTTR did not differ among patients who did versus did not receive a loading dose (data not shown). Also, although there were no differences between the two arms in terms of clinical outcomes like bleeding or thromboembolism, these outcomes were secondary and our study was not powered to detect possible differences. Lastly, most of our patients had a target INR of 2–3, which may limit the generalizability of the study to this population.

## CONCLUSION

Our study demonstrates that CWD is not superior to FWD in improving PTTR, anticoagulation quality measures, and clinical outcomes. Our results aid in understanding the reasons for the contradicting results of the landmark trials of genotype‐guided warfarin dosing. Future prospective studies evaluating this research question in more diverse populations may be warranted to validate these results.

## AUTHOR CONTRIBUTIONS

All authors wrote the manuscript. A.M.F., A.E.B., H.E., and M.O.S. designed the research, performed the research, and analyzed the data.

## FUNDING INFORMATION

Grant: IRGC‐06‐JI‐19‐205 Hamad Medical Corporation (Qatar).

## CONFLICT OF INTEREST STATEMENT

The authors declared no competing interests in this work.

## Supporting information


TableS1.

TableS2.
