# Supplementary material for: Clinical versus fixed warfarin dosing and the impact on quality of anticoagulation (The ClinFix trial)
Source: Clin Transl Sci. 2024 Jun 10;17(6):e13797. doi: 10.1111/cts.13797 (PMC11164972; doi:10.1111/cts.13797)
Supplement: Supplementary file 1 — TableS1. [file CTS-17-e13797-s002.docx]

Table S1. Interacting medications details and their interaction category

| **Interacting Medication** | **Interaction Category**^a^ |
| --- | --- |
| **CWD Arm** | |
| Amiodarone (1 patient CWD) | D |
| Amoxicillin/Clavulanic acid (3 patient CWD) | C |
| Carbamazepine (2 patient CWD) | D |
| Ciprofloxacin (1 patient CWD) | C |
| Fenofibrate (1 patient CWD) | D |
| Fluconazole (1 patient CWD) | D |
| Prednisolone (1 patient CWD) | C |
| **FWD Arm** | |
| Amoxicillin/Clavulanic acid (3 patient FWD) | C |
| Azathioprine (1 patient FWD) | C |
| Carbamazepine (2 patient FWD) | D |
| Carbimazole (3 patient FWD) | C |
| Cefuroxime (1 patient FWD) | C |
| Ciprofloxacin (1 patient FWD) | C |
| Phenytoin (1 patient FWD) | C |
| Prednisolone (3 patient FWD) | C |
| Oral Contraceptive pills (1 patient FWD) | C |

a Interacting category: Definitions according to Lexicomp® interaction checker: Category C: Data demonstrates that this drug can interact with warfarin in a clinically significant matter. The benefits of concomitant use of this medication with warfarin may outweigh the risks. An appropriate monitoring plan should be implemented to identify potential negative effects. Dosage adjustments if one or both agents may be needed in some patients. Category D: Data demonstrates that this drug can interact with warfarin in a clinically significant matter. A patient specific assessment must be conducted to determine whether the benefits of concomitant use of this medication with warfarin may outweigh the risks. Specific actions must be taken in order to realize the benefits and/or minimize the risks from concomitant use of this agent with warfarin. These actions may include aggressive monitoring, empiric dosage changes, or choosing alternative agents.
