# Supplementary material for: Clinical versus fixed warfarin dosing and the impact on quality of anticoagulation (The ClinFix trial)
Source: Clin Transl Sci. 2024 Jun 10;17(6):e13797. doi: 10.1111/cts.13797 (PMC11164972; doi:10.1111/cts.13797)
Supplement: Supplementary file 2 — TableS2. [file CTS-17-e13797-s001.docx]

| **Variables included in the multivariable linear regression** | ***P* value** |
| --- | --- |
| History of Coronary Artery Disease | 0.146 |
| Having Heart Failure | 0.188 |
| Interacting medications ^a^ | 0.185 |

Table S2 Variables included in the multivariable regression analysis

a Interacting medications: Any medication that interacts with warfarin and has a category higher than category C according to Lexicomp® interaction checker.
